# Supplementary material for: Immune Response after COVID-19 mRNA Vaccination in Multiple Sclerosis Patients Treated with DMTs
Source: Biomedicines. 2022 Nov 24;10(12):3034. doi: 10.3390/biomedicines10123034 (PMC9775192; doi:10.3390/biomedicines10123034)
Supplement: Supplementary file 1 [file biomedicines-10-03034-s001.zip › TABLE S1.pdf]

SUPPLEMENTARY MATERIAL

Table S1. ***Inflammatory mediators levels pre-and post-vaccination: differences in each IS-DMT group***

**Cladribine-treated pwMS**		***p***
GrB_pre-vaccination (pg/ml)	GrB_post-vaccination (pg/ml)	
9.876 ± 5.022	9.309 ± 3.448	0.642
IFN-γ_pre-vaccination (pg/ml)	IFN-γ_post-vaccination (pg/ml)	
0.634 ± 0.544	0.580 ± 0.447	0.984
TNF-α _pre-vaccination (pg/ml)	TNF-α_post-vaccination (pg/ml)	
9.133 ± 2.990	9.006 ± 2.050	1.000
**Fingolimod-treated pwMS**		***p***
GrB_pre-vaccination (pg/ml)	GrB_post-vaccination (pg/ml)	
9.587 ± 6.914	9.849 ± 4.728	0.847
IFN-γ_pre-vaccination (pg/ml)	IFN-γ_post-vaccination (pg/ml)	
0.551 ± 0.480	0.778 ± 0.411	0.178
TNF-α _pre-vaccination (pg/ml)	TNF-α_post-vaccination (pg/ml)	
12.513 ± 4.964	11.051 ± 2.980	0.653
**Ocrelizumab-treated pwMS**		***p***
GrB_pre--vaccination (pg/ml)	GrB_post-vaccination (pg/ml)	
10.555 ± 3.264	13.115 ± 4.407	0.021
IFN-γ_pre-vaccination (pg/ml)	IFN-γ_post-vaccination (pg/ml)	
0.986 ± 1.008	0.981 ± 0.865	0.985
TNF-α _pre-vaccination (pg/ml)	TNF-α_post-vaccination (pg/ml)	
10.904 ± 2.258	12.036 ± 4.368	0.311

No significant differences were found in serum GrB, IFN-γ, TNF-α levels between pre-and post-vaccination in each DMTs group (repeated measures ANCOVA).
