# Supplementary material for: Immune Response after COVID-19 mRNA Vaccination in Multiple Sclerosis Patients Treated with DMTs
Source: Biomedicines. 2022 Nov 24;10(12):3034. doi: 10.3390/biomedicines10123034 (PMC9775192; doi:10.3390/biomedicines10123034)
Supplement: Supplementary file 1 [file biomedicines-10-03034-s001.zip › TABLE S2.pdf]

SUPPLEMENTARY MATERIAL

Table S2. ***Inflammatory mediators levels pre-and post-vaccination: differences between seroconverted (1) and non-seroconverted (0) ocrelizumab-treated pwMS patients.***

**Descriptive**	**GrB_pre (pg/ml)**		**GrB _post (pg/ml)**	
	**0**	**1**	**0**	**1**
Mean	10.069	11.720	13.813	11.440
SD	2.914	4.106	4.645	3.658
Minimum	6.040	5.800	6.240	7.700
Maximum	17.300	15.800	24.900	17.200
	**IFN-γ_pre (pg/ml)**		**IFN-γ _post (pg/ml)**	
	**0**	**1**	**0**	**1**
Mean	0.972	0.986	0.723	1.442
SD	1.143	0.246	0.533	1.205
Minimum	0.270	0.650	0.270	0.560
Maximum	4.650	1.320	2.260	3.120
	**TNF-α _pre (pg/ml)**		**TNF-α _post (pg/ml)**	
	**0**	**1**	**0**	**1**
Mean	10.340	12.637	12.702	11.027
SD	2.263	4.521	4.829	3.035
Minimum	6.560	8.250	6.490	6.900
Maximum	13.900	20.100	27.000	15.300

Among the 43 patients tested with ELLA, o-pwMS non-seroconverted (n=13) showed GrB serum levels increase after vaccination (mean ± SD: 13.813 ± 4.645 pg/ml) compared to before (10.069 ± 2.914 pg/ml), while no difference was observed in seroconverted o-pwMS. No differences were found in TNF-α (although a mild trend to increase was evident) and IFN-γ serum levels pre- and post-vaccination (Student's paired t-test).
