# Supplementary material for: Immune Response after COVID-19 mRNA Vaccination in Multiple Sclerosis Patients Treated with DMTs
Source: Biomedicines. 2022 Nov 24;10(12):3034. doi: 10.3390/biomedicines10123034 (PMC9775192; doi:10.3390/biomedicines10123034)
Supplement: Supplementary file 1 [file biomedicines-10-03034-s001.zip › TABLE S3.pdf]

SUPPLEMENTARY MATERIAL

Table S3. ***Lymphocytes immunophenotype pre-and post-vaccination***

Lymphocytes/ul	Pre- vaccination	Post-vaccination	p
	mean ± SD (cells count/ul)	mean ± SD (cells count/ul)	
**CD19+ B cell**			
Cladribine	81.616 ± 77.455	93.874 ± 75.597	1.000
Ocrelizumab	7.401 ± 17.658	7.573 ± 16.432	1.000
**CD3+ T cell**			
Cladribine	811.905 ±496.021	768.905 ± 421546	0.998
Ocrelizumab	1.217.126 ± 527.237	1.305.160 ± 593.981	0.759
**CD4+ T cell**			
Cladribine	475.524 ± 284.689	464.619 ± 320.932	1.000
Ocrelizumab	837.833 ± 394.981	910.143 ± 451.069	0.436
**CD8+ T cell**			
Cladribine	299.905 ± 241.424	286.333 ± 207.881	1.000
Ocrelizumab	379.293 ± 225.432	395.017 ± 256.120	0.898

No differences were found considering lymphocytes count pre-vaccination and post-vaccination both in c-pwMS and o-pwMS (Student's t-test). However, o-pwMS shown a trend to increase of CD4+ T cells. No sufficient lymphocytes immunophenotype data were available of fingolimod-treated patients pre-vaccination.
